# Changes in the frequency and amount of alcohol intake before and during the COVID-19 pandemic

**DOI:** 10.1093/joccuh/uiae055

**Published:** 2024-10-14

**Authors:** Satsue Nagahama, Bibha Dhungel, Ai Hori, Takehiro Michikawa, Keiko Asakura, Yuji Nishiwaki

**Affiliations:** All Japan Labour Welfare Foundation, Tokyo, Japan; Department of Environmental and Occupational Health, School of Medicine, Toho University, Tokyo, Japan; Population Interventions Unit, Melbourne School of Population and Global Health, The University of Melbourne, Melbourne, Australia; Department of Health Policy, National Centre for Child Health and Development, Setagaya-ku, Tokyo, Japan; Department of Global Public Health, Institute of Medicine, University of Tsukuba, Tsukuba, Ibaraki, Japan; Department of Environmental and Occupational Health, School of Medicine, Toho University, Tokyo, Japan; Department of Preventive Medicine, School of Medicine, Toho University, Tokyo, Japan; Department of Environmental and Occupational Health, School of Medicine, Toho University, Tokyo, Japan

**Keywords:** alcohol, COVID-19, Japan, pandemic, alcohol consumption, consumption patterns

## Abstract

Objectives: Concerns have been raised regarding the impact of the COVID-19 pandemic on alcohol consumption patterns, which can have implications for public health. In this descriptive study, we aimed to show the change in the frequency and amount of alcohol consumption in Japan before and during the COVID-19 pandemic periods.

Methods: We analyzed data from annual health checkups among Japanese workers from April 2018 to March 2021. Changes in the frequency (daily, occasionally, rarely/never) and amount per one-time (4 categories by Japanese alcohol unit) of alcohol consumed among 331 200 participants were summarized by sex as 1-year changes in the periods before (fiscal year [FY] 2018 to FY 2019) and during (FY 2019 to FY 2020) the COVID-19 pandemic.

Results: Among daily drinkers and rarely/never drinkers, overall, 1-year changes in the frequency of alcohol consumption during the pandemic were mostly consistent with changes before the pandemic, for both sexes. The number of occasional drinkers who drank less frequently a year later increased during the pandemic compared with before the pandemic (from 9.6% to 11.6% among men and from 12.9% to 16.5% among women); however, occasional drinkers who drank more frequently showed a small increase. Collectively, both men and women showed a slight decrease in both the frequency and amount of alcohol consumption during the pandemic among occasional drinkers.

Conclusions: No major shifts in alcohol consumption habits occurred during the pandemic in our study population. Occasional drinkers tended to drink less during the pandemic, suggesting that initial concerns about increased alcohol consumption owing to the pandemic were unfounded.

## Teaser

Key points:

- Previous studies showed contradictory results on COVID-19's impact on alcohol consumption, reflecting both increased stress-related intake and decreased consumption due to limited social opportunities.
- This study examined changes in alcohol consumption before and during the COVID-19 pandemic using prospectively collected data.
- Our study identified minimal changes in alcohol consumption during the pandemic for both genders, with occasional drinkers notably showing a decrease in both frequency and amount.

## 1. Background

Alcohol consumption in Japan is a culturally important social activity.^1^ However, concerns have been raised about the impact of the COVID-19 pandemic on drinking habits.^2^^,^^3^ The pandemic started in early 2020 and has greatly impacted global health and society.^4^ The Japanese government declared a state of emergency for 2 months from April 7, 2020,^5^ and 3 more times in several metropolitan areas, up to 2021. During the state of emergency declarations, various safety measures were adopted, including urging people to stay home and discouraging gatherings.^6^ Thus, people had fewer opportunities to go out and they spent more time at home, resulting in concerns about the potential increase in stress and anxiety, and the negative impacts on drinking habits.^7^ In response to these concerns, the World Health Organization and the Japan Medical Association issued information to prevent the worsening of drinking habits.^8^ Stress and anxiety associated with the pandemic may have exacerbated changes in alcohol consumption patterns^9^ and increased the risk of alcohol-related harm and mental health disorders.^10^ Japanese individuals with problematic drinking habits were reportedly more prone to engage in high-risk behaviors during the COVID-19 pandemic,^11^^‑^^13^ with men more likely to drink alcohol than women.^14^ Understanding these changes can provide insights into managing alcohol-related issues in health care settings.

Several previous studies have assessed alcohol consumption patterns during the COVID-19 pandemic. The findings are mixed; studies published in Europe,^15^^‑^^17^ the United States,^18^^‑^^20^ and other countries have reported varying changes in alcohol consumption patterns during the pandemic. In some cases, there was an increase in alcohol consumption owing to stress, anxiety, and as a coping mechanism.^21^^‑^^23^ However, other studies have reported a decrease in the consumption of alcohol owing to reduced social opportunities, financial challenges, and health concerns.^24^^‑^^29^ A study conducted among the Japanese population reported a decrease in binge drinking during the pandemic.^30^ However, that study relied on a small online sample and retrospectively assessed self-reported data on alcohol consumption before the pandemic.

The magnitude and direction of changes in alcohol consumption during the pandemic may vary across populations, countries, contexts, and time periods. Many previous studies were conducted after the onset of the pandemic and may therefore have introduced bias owing to retrospective collection of self-reported data.^31^^‑^^33^ Definitive conclusions are difficult to draw from these studies because there are no prospectively collected pre- and post-pandemic data. To understand the impact of the COVID-19 pandemic on alcohol consumption patterns, the frequency and pattern of alcohol consumption should be followed before the pandemic period. Such observations would be helpful to inform public health strategies and interventions to mitigate potential harms associated with alcohol use during a public health crisis.

The objective of this descriptive study was to clarify the frequency and pattern of alcohol consumption in Japan before and during the COVID-19 pandemic.

## 2. Methods

### 2.1. Study population

**Figure 1:**
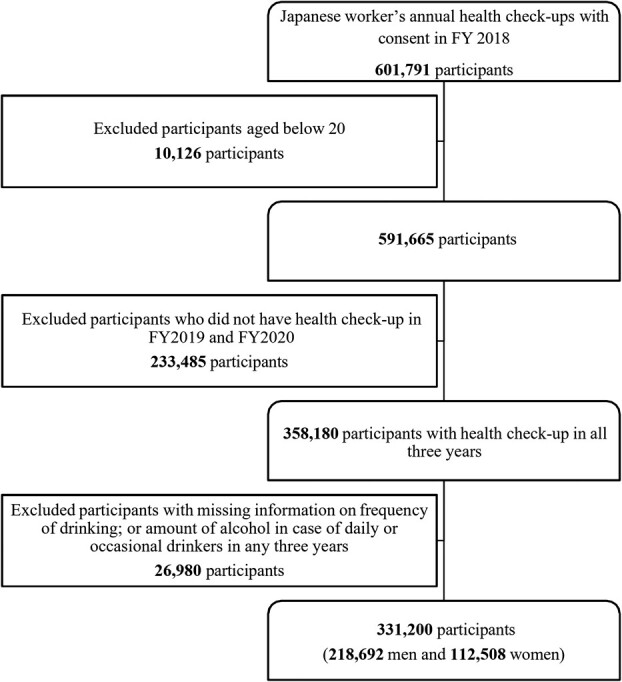
Flow of data extraction. FY, fiscal year.

This follow-up study used data from Japanese workers’ annual health checkups, which are mandatory for all workers under the Industrial Safety and Health Act. The All-Japan Labour Welfare Foundation provided data from April 2018 through March 2021 from its health checkup centers in 9 prefectures of eastern Japan (Aomori, Nagano, Yamagata, Ibaraki, Gunma, Tokyo, Kanagawa, Nagano, Mie). As a result, the participants were mainly employees from eastern Japan. Figure 1 shows the flow of data extraction. We excluded data for which consent to participate was not given, resulting in 601 791 participants in fiscal year 2018 (FY 2018). The participants were mainly Japanese employees, with a small number of their dependents, employers, and foreign workers. A total of 358 180 participants aged over 20 years received a health checkup each year from FY 2018 to FY 2020. Of these, we excluded participants with missing information on the frequency of drinking or the amount of alcohol consumed per drink for daily and occasional drinkers (11 478 participants in FY 2018, 25 591 participants in FY 2019, and 10 314 participants in FY 2020). After excluding 26 980 participants with missing data for 1 or more years, we finally included 331 200 participants (218 692 men and 112 508 women) in the analysis.

**Table 1:** Characteristics of study participants (men, *n* = 218 692; women, *n* = 112 508).

	**Men**			**Women**		
	**FY 2018**	**FY 2019**	**FY 2020**	**FY 2018**	**FY 2019**	**FY 2020**
**Age, y, mean (SD)**	44.4 (12.9)	45.4 (13.0)	46.5 (12.9)	45.1 (13.2)	46.1 (13.2)	47.1 (13.1)
**BMI, kg/m** ^**2**^ **, mean (SD)**	23.8 (3.9)	23.9 (3.9)	24.1 (4.0)	22.2 (4.1)	22.3 (4.1)	22.4 (4.2)
**Alcohol consumption, *n* (%)**						
**Rarely or never drinkers**	71 049 (32.5)	71 792 (32.8)	74 571 (34.1)	61 138 (54.3)	61 398 (54.6)	63 720 (56.6)
**Occasional drinkers**	77 064 (35.2)	76 019 (34.8)	71 816 (32.8)	37 632 (33.5)	37 368 (33.2)	34 582 (30.7)
**Daily drinkers**	70 579 (32.3)	70 881 (32.3)	72 305 (33.1)	13 738 (12.2)	13 742 (12.2)	14 206 (12.6)
**Smoking status, *n* (%)**						
**Nonsmoker**	83 759 (38.3)	83 103 (38)	82 228 (37.6)	87 194 (77.5)	87 194 (77.5)	87 081 (77.4)
**Ex-smoker**	40 895 (18.7)	43 738 (20)	47 675 (21.8)	8663 (7.7)	9113 (8.1)	10 013 (8.9)
**Consuming ≤20 cigarettes/day**	75 230 (34.4)	73 699 (33.7)	72 168 (33)	15 864 (14.1)	15 414 (13.7)	14 739 (13.1)
**Consuming >20 cigarettes/day**	18 808 (8.6)	18 151 (8.3)	16 621 (7.6)	788 (0.7)	788 (0.7)	675 (0.6)
**Walking time, ≥60 min/d, *n* (%)**	94 256 (43.1)	95 568 (43.7)	96 224 (44)	40 953 (36.4)	41 740 (37.1)	41 290 (36.7)
**Diabetes,** ^**a**^ ***n* (%)**	12 465 (5.7)	12 684 (5.8)	13 778 (6.3)	3375 (3.0)	3263 (2.9)	3488 (3.1)
**Hypertension,** ^**b**^ ***n* (%)**	44 832 (20.5)	47 456 (21.7)	53 361 (24.4)	14 401 (12.8)	15 301 (13.6)	18 226 (16.2)
**Dyslipidemia,** ^**c**^ ***n* (%)**	65 826 (30.1)	66 701 (30.5)	70 200 (32.1)	18 901 (16.8)	19 351 (17.2)	20 026 (17.8)

Abbreviations: BMI, body mass index; FY, fiscal year.

a Glycated hemoglobin (HbA1c) ≥6.5% or receiving medication.

b Systolic blood pressure ≥140 mmHg, diastolic blood pressure ≥90 mmHg, or receiving medication.

c Triglyceride level ≥150 mg/dL (1.7 mmol/L), high-density lipoprotein cholesterol <40 mg/dL (1.04 mmol/L) in men and <50 mg/dL (1.3 mmol/L) in women, or receiving medication.

### 2.2. Ascertainment of alcohol frequency and amount

We used a self-administered questionnaire developed by the Ministry of Health, Labour, and Welfare of Japan to assess the frequency of alcohol consumption and the amount per one-time intake for participants undergoing a specific health examination, as part of the national health checkup system for metabolic syndrome.^34^ The questionnaire was used to assess the frequency of alcohol consumption, which was reported as either “none or rarely,” “occasionally,” or “daily drinking.” The one-time intake was reported as <1 gou, 1 to <2 gou, 2 to <3 gou, or ≥3 gou per day. One gou of sake, a traditional Japanese beverage, is equivalent to approximately 180 mL of 10% to 14% ethanol and contains approximately 23 g of ethanol.^35^ For each drinking habit, respondents were asked to indicate their status at the time of the health checkup visit. The questionnaire did not require a response for drinkers with responses of “none or rarely,” and thus we analyzed the amount for daily and occasional drinkers only.

### 2.3. Statistical analysis

We summarized data using Stata SE version 16 (StataCorp LLC, College Station, TX, USA) to calculate frequency and percent for alcohol consumption frequency and amount. We created summary graphs separately for men and women and stratified by year, to provide insight into alcohol consumption patterns among different groups according to sex and year.

Mean and SD of the participants’ characteristics were calculated by gender for age (years) and body mass index (kg/m^2^). Proportions were calculated for each category of alcohol consumption (none or rarely, occasionally, or daily drinking), smoking status (nonsmoker, ex-smoker, daily consuming ≤20 cigarettes/day, or daily consuming>20 cigarettes/day), diabetes (HbA1c ≥6.5 % according to National Glycohemoglobin Standardization Program standard or receiving medication), hypertension (systolic blood pressure ≥140 mmHg, diastolic blood pressure ≥90 mmHg or receiving medication), and dyslipidemia (triglyceride level ≥150 mg/dL [1.7 mmol/L]; high-density lipoprotein cholesterol level <40 mg/dL [1.04 mmol/L] in men and <50 mg/dL [1.3 mmol/L] in women, or receiving medication). To examine the one-year change in frequency of alcohol consumption before (FY2018 to FY2019) and during (FY2019 to FY2020) the pandemic, the percentages of frequency of alcohol consumption in FY2019 and FY2020 were calculated for frequency of alcohol consumption in FY2018 and FY2019, respectively. The percentages of amount were calculated in the same way for occasional and daily drinkers in the base year. For participants who reported drinking occasionally, a group with significant changes in drinking habits in the following year, characteristics in 2018 and 2019 were presented for each category of drinking frequency in the following year. The trends in participants' drinking habits and the number of health checkup attendees, along with the incidence of COVID-19 cases in Japan, are graphically depicted based on percentages across health checkup months.

## 3. Results

The characteristics of the participants are presented in Table 1. The mean (SD) age of the included participants in 2018 was 44.4 (12.9) years in men, and 45.1 (13.2) years in women. The proportion of current nonsmokers increased slightly for both men and women from 2018 to 2020. Table 2 shows the frequency and amount of alcohol consumption by men and women from FY 2018 to FY 2020. Throughout the 3 years, a roughly equal proportion of men reported consuming alcohol daily, occasionally, and rarely, with each category accounting for approximately one-third of the total men. Among men, there were marginal variations in the amount of alcohol consumed per drink during these periods: <1 gou (33.6%, 33.2%, 34.9% of the total, respectively), 1 to <2 gou (43.5%, 43.8%, 43.8%, respectively), and 2 to <3 gou (6.0%, 6.0%, 5.1%, respectively). In contrast, more than half of women reported rarely or never consuming alcohol. When women did consume alcohol, most (approximately 60%) drank less than 1 gou.

**Table 2:** Frequency of alcohol consumed from FY 2018 to FY 2020 among men (*n* = 218 692) and women (*n* = 112 508).

	**FY 2018**	**FY 2019**	**FY 2020**
***Men***			
**Alcohol frequency, *n* (%)**			
**Daily**	70 579 (32.3)	70 881 (32.3)	72 305 (33.1)
**Occasional**	77 064 (35.2)	76 019 (34.8)	71 816 (32.8)
**Rarely or never**	71 049 (32.5)	71 792 (32.8)	74 571 (34.1)
**Amount per day,** ^**a**^ ***n* (%)**			
**<1 gou** ^**b**^	50 341 (33.6)	49 495 (33.2)	51 066 (34.9)
**1-2 gou**	65 186 (43.5)	65 384 (43.8)	64 102 (43.8)
**2-3 gou**	25 331 (16.9)	25 296 (17.0)	23 723 (16.2)
**≥3 gou**	8937 (6.0)	8962 (6.0)	7463 (5.1)
***Women***			
**Alcohol frequency, *n* (%)**			
**Daily**	13 738 (12.2)	13 742 (12.2)	14 206 (12.6)
**Occasional**	37 632 (33.5)	37 368 (33.2)	34 582 (30.7)
**Rarely or never**	61 138 (54.3)	61 398 (54.6)	63 720 (56.6)
**Amount per day,** ^**a**^ ***n* (%)**			
**<1 gou**	29 711 (56.6)	29 360 (56.1)	29 683 (58.8)
**1-2 gou**	17 570 (33.5)	17 700 (33.9)	16 527 (32.8)
**2-3 gou**	4162 (7.9)	4183 (8.0)	3392 (6.7)
**≥3 gou**	1041 (2.0)	1050 (2.0)	844 (1.7)

Abbreviation: FY, fiscal year.

a Contains missing data for non- or infrequent drinkers.

b One gou equals 23 g of ethanol.

Table S1 shows that occasional drinkers who reduced their drinking frequency in the following year tended to have a lower mean age and comprised a higher proportion of nonsmokers and people with hypertension and dyslipidemia than those who increased their drinking frequency. This trend was consistent in the year before the COVID-19 pandemic (2018 to 2019) and the year spanning the onset of the COVID-19 pandemic (2019 to 2020). Figure S1 shows the alcohol consumption habits of individuals attending health checkups each month. Compared with previous years, the number of attendees decreased in April and May 2020 during the COVID-19 pandemic, whereas it increased in August and September 2020. There was little impact observed on alcohol consumption habits attributable to the COVID-19 spread. Throughout the 3 years of 2018 to 2020, the proportion of daily drinkers was higher in April than in other months, whereas the proportion of nondrinkers was higher from December to March compared with other months.

**Figure 2:**
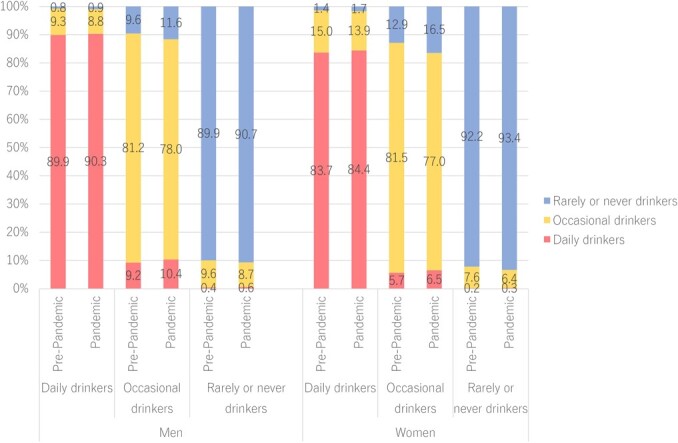
Change in frequency of drinking in the following year, before the pandemic and during the pandemic. The bar shows frequency after 1 year. Pre-pandemic means changes from 2018 to 2019, and pandemic refers to changes from 2019 to 2020.

Figure 2 provides information on the change in alcohol consumption frequencies among men and women before (between FY 2018 and FY 2019) and during (between FY 2019 and FY 2020) the COVID-19 pandemic. Daily drinkers during the pandemic accounted for 90.3% versus 89.9% before the pandemic for men, and for 84.4% versus 83.7% for women. The proportion of daily drinkers who switched to occasional drinking was 9.3% in the before-pandemic period and 8.8% in the pandemic period among men; these proportions were 15.0% and 13.9% among women. Among daily drinkers, the 1-year changes in the frequency of alcohol consumption before the pandemic were mostly consistent with changes during the pandemic, for both sexes.

**Figure 3:**
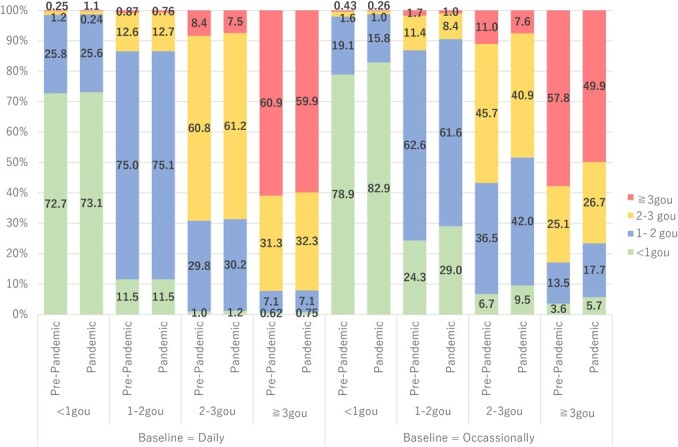
Change in amount of drinking before and during the pandemic at different baseline frequency levels. The bar shows amount after 1 year. Pre-pandemic means changes from 2018 to 2019, and pandemic refers to changes from 2019 to 2020. One gou equals 23 g of ethanol.

Figure 3 depicts information on the amount of alcohol consumed with different baseline frequencies among the total population. We did not observe sex differences regarding the pattern of alcohol consumption (see Table S2). Among people who drank daily in the baseline year, changes in the amount of alcohol consumed were similar in the periods before and during the pandemic. However, among those who drank occasionally at baseline, the amount of alcohol consumed decreased during the pandemic compared with that before the pandemic. For example, the proportion of occasional drinkers who continuously consumed more than 3 gou of alcohol per one-time intake decreased from 57.8% before the pandemic to 49.9% during the pandemic.

## 4. Discussion

Alcohol consumption patterns during the COVID-19 pandemic remained relatively unchanged compared with the before-pandemic period for both men and women in our study. Additionally, there was a decrease in the frequency and amount of alcohol consumption among occasional drinkers. These findings suggest that initial concerns about worsening alcohol habits owing to the pandemic were unfounded. Our findings align with longitudinal studies conducted in Europe^15^^‑^^17^ and the United States,^18^^‑^^20^ as well as a previous Japanese study,^14^ which consistently demonstrate a lack of significant changes or even a decrease in drinking habits during the pandemic.

Daily drinkers did not exhibit substantial changes in their drinking habits in response to the COVID-19 pandemic, as highlighted by our findings. However, among occasional drinkers, there was an increase in the frequency of never or rarely drinking and a reduction in the amount of alcohol consumed in the following year during the pandemic period, compared with the before-pandemic period. Occasional drinkers often include individuals who consume alcohol for social reasons, who may have been influenced by the decreased opportunities for socializing as well as self-restraint measures implemented during the pandemic. The Japanese government asked pub or restaurant owners not to provide alcoholic beverages during the state of emergency declaration and offered monetary compensation for doing so. Nevertheless, there was concern that some occasional drinkers may compensate for the lack of social settings by increasing their alcohol consumption at home, especially if they perceive alcohol as a coping mechanism during stressful times. Several studies^14^^,^^17^^,^^25^^‑^^27^^,^^29^^,^^30^^,^^32^^,^^33^ and meta-analyses^28^ along with other publications have reported a decrease in overall alcohol consumption, which is consistent with the findings among occasional drinkers in our study. Notably, however, most of those studies were cross-sectional online surveys, with data collected after the pandemic. Conversely, some studies have reported an increase in alcohol consumption during the COVID-19 pandemic.^13^^,^^16^^,^^23^ The study findings also raise important considerations regarding the potential influence of broader societal trends on drinking behavior. Although the COVID-19 pandemic made little difference on alcohol consumption patterns during the pandemic compared with previous years, reflected by the graph confirming alcohol consumption habits by month of health checkup, it is necessary to contextualize these findings within the broader landscape of alcohol consumption trends in Japan. It is noted that alcohol consumption in Japan has exhibited a gradual decline over the years. The annual alcohol consumption per adult decreased from its peak of 101.8 L in 1992 to 78.2 L in 2019.^36^ This long-term trend underscores the importance of considering pre-existing patterns of alcohol consumption when interpreting our results. The observed differences between the pre-pandemic and pandemic periods may reflect not only the impact of the pandemic itself but also ongoing shifts in societal norms and behaviors related to alcohol consumption.

Cultural differences are highlighted in studies comparing pandemic responses between European and Asian countries, noting that the Japanese tend to be more group-oriented and often exhibit similar behaviors to those around them.^37^^,^^38^ A German study, which used data before the COVID-19 pandemic, reported a return of alcohol consumption to pre-lockdown levels after lockdown measures were lifted.^32^ However, we did not observe a similar trend, suggesting that cultural aspects of Japanese society may have contributed to more prolonged self-restraint in drinking compared with populations in other countries, such as Germany. Further research is needed to explore and better understand these cultural differences and their implications on drinking behaviors during a public health crisis, such as a pandemic.

We did not find compelling evidence of an increase in risky drinking. Daily drinkers maintained consistent alcohol consumption levels after the pandemic, and the percentage of occasional drinkers who consistently consumed more than 3 gou of alcohol after 1 year decreased during the pandemic period compared with the pre-pandemic period. Previous studies have reported a decrease in binge drinking^30^ whereas others have reported an increase.^12^^,^^14^^,^^16^^,^^39^ Notably, however, those cross-sectional studies were conducted using retrospective data collected after the COVID-19 pandemic.^12^^,^^14^^,^^16^^,^^39^

### 4.1. Strengths and limitations

To the best of our knowledge, this was the first study with a large sample size and using pre-pandemic surveys conducted in Japan to examine alcohol consumption patterns, which strengthens the validity of our findings. Data on alcohol consumption in this study were collected using the same questionnaire at the time of health examination visits both before and during the pandemic. On the other hand, most previous studies have been cross-sectional and collected previous information after the pandemic. Due to these differences in data collection, direct comparisons with previous studies are challenging, but we consider that this study contributes additional evidence regarding the changes in drinking habits brought about by the COVID-19 pandemic.

Despite these strengths, this study had certain limitations. Because the data in this study are from the Japanese population, caution is needed in generalizing the findings to other populations. Secondly, during the first state of emergency in April and May 2020 in Japan, the Ministry of Health, Labour and Welfare requested the postponement of health checkups. As a result, fewer people underwent health checkups during that time, so potentially the impact of restrictions on leaving the house in the early phase of the COVID-19 pandemic may have been missed. Similarly, the association between the pandemic and alcohol consumption habits may be influenced by socioeconomic status.^14^^,^^18^ Unfortunately, we lacked information on socioeconomic factors such as mental health or economic stress in this study, which limited our ability to thoroughly examine this aspect. Additionally, the potential of measurement bias due to self-reported alcohol consumption is an essential consideration in interpreting the findings of this study. Many previous studies have reported inconsistencies in the accuracy of self-reported alcohol consumption.^40^^,^^41^ The potential underreporting of alcohol consumption necessitates careful interpretation of the data. Finally, the analysis focused on individuals who underwent health examinations for 3 consecutive years, resulting in the exclusion of those who retired during the pandemic. Nonetheless, considering that the overall number of health examinations per year did not decrease significantly, the impact is presumed to be minimal.

These strengths and limitations should be taken into consideration when interpreting the findings of this study. Further research is needed to address these limitations and provide a more comprehensive understanding of the impact of the pandemic on alcohol consumption habits in Japan.

## 5. Conclusion

The present research sheds light on the immediate effects of the COVID-19 pandemic on drinking behaviors. Our findings indicate that there were minimal differences regarding changes in alcohol consumption owing to the pandemic, both in terms of frequency and quantity, in both men and women. Additional research is needed to comprehensively examine the long-term effects of the pandemic on drinking habits, including cultural variations and the implications of these findings in diverse populations and contexts.

## Supplementary Material

Web_Material_uiae055
