# Supplementary material for: Changes in the frequency and amount of alcohol intake before and during the COVID-19 pandemic
Source: J Occup Health. 2024 Oct 14;66(1):uiae055. doi: 10.1093/joccuh/uiae055 (PMC11561262; doi:10.1093/joccuh/uiae055)
Supplement: Web_Material_uiae055 [file web_material_uiae055.zip › â~+Sup1. T1.pdf]

Table S1. Characteristics of occasional drinker by changes in drinking frequency after 1 year.

| | 2018 to 2019 | | | 2019 to 2020 | | |
|---|---|---|---|---|---|---|
| | Decreased | Unchanged | Increased | Decreased | Unchanged | Increased |
| Number | 12,216 | 93,236 | 9,244 | 14,993 | 88,068 | 10,326 |
| Women (%) | 39.7 | 32.9 | 23.0 | 41.1 | 32.7 | 23.7 |
| Age (years)[a] | 39.9 ± 13.3 | 41.1 ± 13.0 | 44.5 ± 12.5 | 40.9 ± 13.4 | 42.3 ± 13.0 | 45.0 ± 12.4 |
| BMI (kg/m2)[a] | 23.3 ± 4.3 | 23.3 ± 4.1 | 23.2 ± 3.6 | 23.4 ± 4.4 | 23.4 ± 4.1 | 23.3 ± 3.8 |
| Smoking status, (%) | | | | | | |
| Non-smoker | 59.0 | 55.1 | 38.8 | 60.7 | 54.7 | 40.0 |
| Ex-smoker | 11.6 | 14.3 | 21.3 | 11.9 | 15.6 | 21.0 |
| Daily consuming ≤20 cigarettes/day | 25.0 | 26.6 | 34.4 | 23.4 | 25.8 | 33.2 |
| Daily consuming >20 cigarettes/day | 4.4 | 4.1 | 5.6 | 4.0 | 3.9 | 5.8 |
| Walking time, ≥60 min/day, (%) | 44.0 | 43.1 | 42.4 | 43.7 | 43.3 | 43.0 |
| Diabetes, (%) [b] | 4.5 | 4.0 | 4.5 | 3.9 | 4.0 | 4.3 |
| Hypertention, (%) [c] | 12.8 | 14.0 | 18.8 | 12.9 | 15.3 | 19.7 |
| Dyslipidemia, (%) [d] | 23.8 | 23.3 | 25.1 | 23.7 | 24.0 | 24.8 |

[a] Mean ± SD

[b] HbA1c ≥6.5 or receiving medication.

[c] Systolic blood pressure ≥140mmHg, diastolic blood pressure ≥90 mmHg or receiving medication.

[d] Triglyceride level ≥150mg/dL (1.7mmol/L), high-density lipoprotein cholesterol level <40 mg/dL (1.04 mmol/L) in men and <50 mg/dL (1.3 mmol/L) in women or receiving medication.
