# Supplementary material for: Changes in the frequency and amount of alcohol intake before and during the COVID-19 pandemic
Source: J Occup Health. 2024 Oct 14;66(1):uiae055. doi: 10.1093/joccuh/uiae055 (PMC11561262; doi:10.1093/joccuh/uiae055)
Supplement: Web_Material_uiae055 [file web_material_uiae055.zip › â~+Sup3 T2.pdf]

Supplementary file

Table S2. Change in amount of drinking during pre-pandemic period and pandemic period

**Men (n = 218,692)**									
**At base=daily**									
	**Pre-pandemic period**					**Pandemic period**			
	**FY 2019**					**FY 2020**			
**FY 2018**	**<1 gou**	**1-2 gou**	**2-3 gou**	**≥3 gou**	**FY 2019**	**<1 gou**	**1-2 gou**	**2-3 gou**	**≥3 gou**
**Alcohol amount**					**Alcohol amount**				
<1 gou	11,696 (71.1)	4,493 (27.3)	213 (1.3)	48 (0.29)	<1 gou	11,711 (71.6)	4,411 (27.0)	184 (1.1)	49 (0.3)
1-2 gou	3,799 (10.6)	26,963 (75.4)	4,663 (13.1)	317 (0.89)	1-2 gou	3,891 (10.8)	27,060 (75.2)	4,743 (13.2)	275 (0.76)
2-3 gou	130 (0.9)	4,226 (29.2)	8,894 (61.5)	1,214 (8.4)	2-3 gou	160 (1.1)	4,280 (29.5)	8,958 (61.8)	1,098 (7.6)
≥3 gou	22 (0.64)	231 (6.76)	1,060 (31.0)	2,102 (61.6)	≥3 gou	25 (0.72)	234 (6.8)	1,111 (32.2)	2,084 (60.3)
**At base=occasional**									
	**Pre-pandemic period**					**Pandemic period**			
	**FY 2019**					**FY 2020**			
**FY 2018**	**<1 gou**	**1-2 gou**	**2-3 gou**	**≥3 gou**	**FY 2019**	**<1 gou**	**1-2 gou**	**2-3 gou**	**≥3 gou**
**Alcohol amount**					**Alcohol amount**				
<1 gou	21,051 (76.1)	5,899 (21.3)	564 (2.0)	159 (0.57)	<1 gou	21,221 (80.4)	4,762 (18.0)	332 (1.3)	86 (0.33)
1-2 gou	6,135 (22.5)	17,310 (63.4)	3,305 (12.1)	544 (2.0)	1-2 gou	7,043 (26.5)	16,758 (63.1)	2,443 (9.2)	295 (1.1)
2-3 gou	657 (6.4)	3,613 (35.1)	4,801 (46.6)	1,222 (11.9)	2-3 gou	876 (8.8)	4,039 (40.5)	4,257 (42.7)	807 (8.1)

≥3 gou	177 (3.4)	680 (13.0)	1,272 (24.3)	3102 (59.3)	≥3 gou	281 (5.5)	852 (16.7)	1,366 (26.7)	2,618 (51.2)
**Women (n = 112,508)**									
**At base=daily**									
	**Pre-pandemic period**					**Pandemic period**			
	**FY 2019**					**FY 2020**			
**FY 2018**	**<1 gou**	**1-2 gou**	**2-3 gou**	**≥3 gou**	**FY 2019**	**<1 gou**	**1-2 gou**	**2-3 gou**	**≥3 gou**
**Alcohol amount**					**Alcohol amount**				
<1 gou	4,421 (77.4)	1,224 (21.4)	62 (1.1)	8 (0.14)	<1 gou	4,415 (77.2)	1,248 (21.8)	50 (0.87)	4 (0.07)
1-2 gou	1,024 (16.7)	4,458 (72.6)	612 (10.0)	46 (0.75)	1-2 gou	959 (15.7)	4,528 (74.0)	588 (9.6)	44 (0.72)
2-3 gou	24 (1.7)	513 (36.2)	763 (53.9)	116 (8.2)	2-3 gou	29 (2.0)	520 (36.6)	777 (54.7)	95 (6.7)
≥3 gou	1 (0.35)	32 (11.3)	99 (34.9)	152 (53.5)	≥3 gou	3 (1.1)	32 (11.9)	90 (33.3)	145 (53.7)
**At base=occasional**									
	**Pre-pandemic period**					**Pandemic period**			
	**FY 2019**					**FY 2020**			
**FY 2018**	**<1 gou**	**1-2 gou**	**2-3 gou**	**≥3 gou**	**FY 2019**	**<1 gou**	**1-2 gou**	**2-3 gou**	**≥3 gou**
**Alcohol amount**					**Alcohol amount**				
<1 gou	15,988 (82.9)	3,053 (15.8)	200 (1.0)	44 (0.23)	<1 gou	15,704 (86.6)	2,293 (12.6)	115 (0.6)	28 (0.15)
1-2 gou	3,056 (29.1)	6,330 (60.3)	993 (9.5)	114 (1.1)	1-2 gou	3,633 (35.3)	5,922 (57.5)	663 (6.4)	79 (0.77)
2-3 gou	211 (8.2)	1,087 (42.1)	1,085 (42.0)	199 (7.7)	2-3 gou	314 (12.7)	1,199 (48.3)	834 (33.6)	135 (5.4)
≥3 gou	34 (4.8)	125 (17.6)	220 (30.9)	332 (46.7)	≥3 gou	50 (7.1)	179 (25.5)	190 (27.0)	284 (40.4)

Table S2 shows the change in the amount of alcohol consumption over two years during the pre-pandemic (FY 2018-FY 2019) and the corona pandemic period (FY 2019-FY 2020), separately by baseline levels of alcohol consumption. For both men and women who drank daily at baseline, there was no apparent difference in alcohol consumption between pre-pandemic and pandemic periods. Among men and women who drank occasionally at baseline, there was a greater decrease in alcohol consumption over the two years during the pandemic period compared to the pre-pandemic period.
