# Supplementary figures and images for: Changes in the frequency and amount of alcohol intake before and during the COVID-19 pandemic
Source: J Occup Health. 2024 Oct 14;66(1):uiae055. doi: 10.1093/joccuh/uiae055 (PMC11561262; doi:10.1093/joccuh/uiae055)

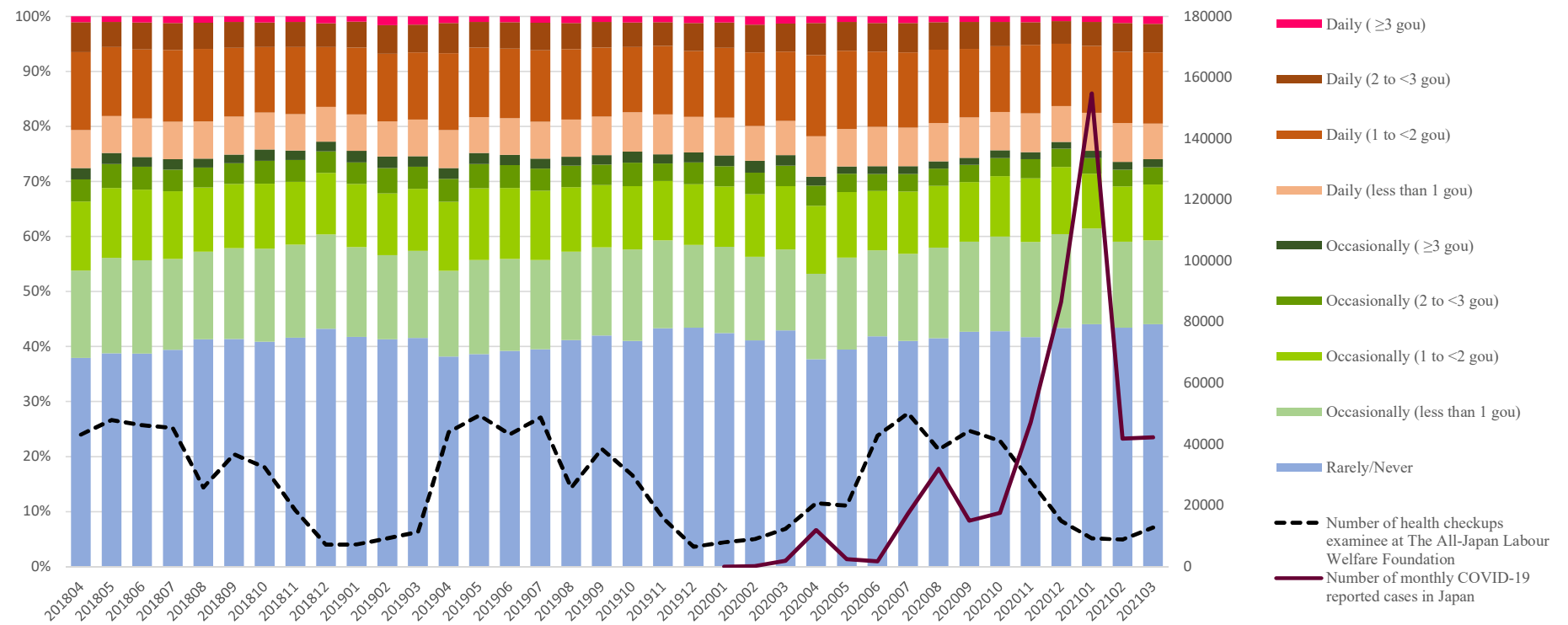

Figure S1. Change in drinking habits by month of consultation of health checkup

Supplement: Web_Material_uiae055 [file web_material_uiae055.zip › â~+Supï1⁄4'.F1_month0613.pdf]
